# An alphavirus replicon particle delivering prefusion-stabilized spike protein provides potent immunoprotection against SARS-CoV-2 Omicron variant

**DOI:** 10.1038/s41392-022-01246-x

**Published:** 2022-12-14

**Authors:** Hong-Qing Zhang, Ya-Nan Zhang, Zhe-Rui Zhang, Xiao-Ling Chen, Yan-Yan Hu, Yu-Jia Shi, Jing Wang, Cheng-lin Deng, Bo Zhang, Xiao-Dan Li, Han-Qing Ye

**Affiliations:** 1 Key Laboratory of Special Pathogens and Biosafety, Wuhan Institute of Virology, Center for Biosafety Mega-Science, Chinese Academy of Sciences, Wuhan, China; 2 University of Chinese Academy of Sciences, Beijing, 100049 China; 3 Hunan Normal University, School of Medicine, Changsha, 410081 China

**Keywords:** Preclinical research, Infectious diseases

**Dear Editor,**

The prolonged outbreak and spread of coronavirus disease 2019 (COVID-19), caused by SARS-CoV-2 poses a great threat to global economic and public health. The protective efficacy of the vaccines based on the spike protein (S) of SARS-CoV-2 has been compromised by the emergence of variants of concern (VOC). The Omicron variant which is designated as the fifth VOC by WHO, has rapidly replaced the Delta variant as the dominant variant worldwide since its emergence in November 2021 in South Africa. The Omicron variant carries more than 30 mutations/deletions/insertion in the S protein as well as the receptor binding domain (RBD). Accumulated evidence has demonstrated that the Omicron variant can largely escape from vaccination, convalescent sera, and most of the approved therapeutic monoclonal antibodies,^1^ posing a high risk of infection and reinfection in highly vaccinated populations. Thus, a targeted vaccine against the Omicron is crucial to contain the spread of the variant.

In this study, we generated a SARS-CoV-2 Omicron variant vaccine using Venezuelan equine encephalitis virus (VEEV) replicon to encode the prefusion-stabilized spike protein of the Omicron variant BA.1. VEEV is a positive-sense, single-stranded RNA virus that belongs to the genus *alphavirus* of the family *Togaviridae*. Compared with the conventional non-replicating mRNA, the alphavirus-derived self-amplifying replicon RNA (saRNA) encodes the viral replicase as well as a gene of interest (GOI), which enables the RNA replication and GOI expression in the cytoplasm^2^ and circumvents the risk of genomic integration, thus enhances the safety profile of the saRNA-based vaccines. Additionally, the replicable saRNA could activate an innate immune response, which has the potential to provide an adjuvant effect on vaccine potency. The replicon could be encapsulated into single-round infectious alphavirus replicon particles (VRPs) by in trans supplement of the viral structural proteins and thereafter be delivered into cells by VRP infection.^2^ The alphavirus VRP system has been extensively used in cancer therapy and vaccine development with well-demonstrated safety and tolerability in numerous clinical trials.^2^

The S protein with two prefusion stabilizing K986P and V987P mutations capable of conferring enhanced immunogenicity,^3^ was inserted under the control of the subgenomic promoter of VEEV replicon (VEEV-S-2P). After confirming the expression of S protein in the VEEV-S-2P replicon transfected cells (Fig. 1b, c), the replicon was packaged into VRPs (VRP-S-2P) (Fig.1a). The resulting VRP-S-2P was used to infect naïve BHK-21 cells to measure the infectivity and in vitro delivery efficiency of the replicon. A similar expression pattern of S protein was observed in the VRP-infected cells to that in replicon-transfected cells (Fig. 1b, c), suggesting that the self-replicating VEEV-S-2P replicon can be efficiently delivered by VRP.

**Fig. 1:**
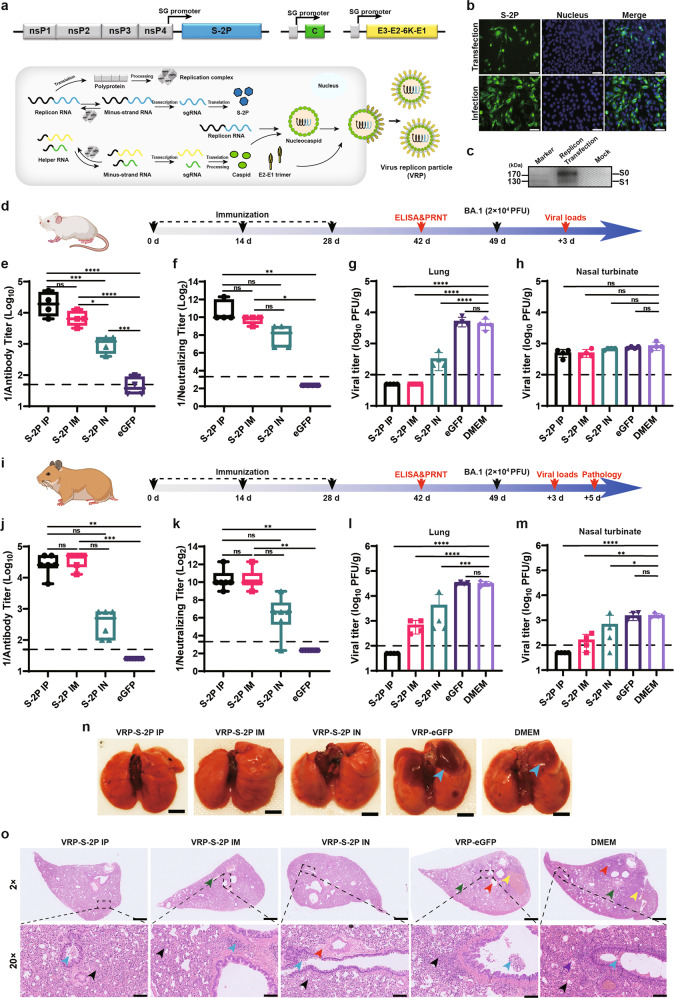
**Development of alphavirus replicon particle vaccine VRP-S-2P against SARS-CoV-2 Omicron variant BA.1. a** Schematic diagram of the VEEV-S-2P replicon and VRP construction. The VEEV-S-2P replicon contains two open reading frames (ORF), encoding four non-structural proteins, ns1–ns4, from Venezuelan equine encephalitis virus (VEEV) and the prefusion-stabilized spike protein of SARS-CoV-2 Omicron variant BA.1. The VEEV-S-2P replicon RNA was co-transfected into BHK cells with two helper RNAs expressing the structural proteins of VEEV, resulting in the production of VRP-S-2P. **b**, **c** Indirect immunofluorescence and western blot detection of SARS-CoV-2 spike protein followed transfection of VEEV-S-2P and infection of VRP-S-2P at an MOI of 1. Scale bar: 50 μm. **d**, **i** The schematic diagram of the VRP-S-2P vaccination in mouse and hamster model. Female Golden Syrian hamsters aged four- to six-weeks and BALB/c aged six- to eight-weeks were immunized with 1×10^6^ FFU VRP-S-2P via intraperitoneal, intramuscular and intranasal routes, respectively. Three immunizations were conducted with two-week intervals. On day 42 post immunization, serum samples were collected. Titers of total RBD-specific IgG (**e**, **j**) were measured by ELISA. **f**, **k** Neutralizing antibody (PRNT_50_ titer) against the Omicron variant BA.1 was determined by PRNT. On day 49 post immunization, the immunized mice and hamsters were challenged intranasally with 2 × 10^4^ PFU Omicron variant BA.1. On day 3 post challenge, mice and hamsters were sacrificed. Viral loads in lungs (**g**, **l**) and nasal turbinates (**h**, **m**) were determined by plaque assay. On day 5 post challenge, hamsters were sacrificed, of which lung tissues were collected. Lung lesions were analyzed by bright field and H&E staining. **n** Representative lungs from indicated groups of hamsters. Severe pathology in the lungs were depicted by blue arrows. Scale bar = 5 mm. **o** Representative H&E staining images from indicated groups of hamsters. Arrows for inflammatory cell infiltration (black), eosinophilic secretions (blue), perivascular edema with lymphocyte infiltration or tissue exudate (red), focal lymphoid infiltration around a few bronchi (purple), alveolar hemorrhage (yellow), and lymphocytes infiltration in a sleeve shape and necrotic exfoliated epithelial cells (green) were marked respectively in the images. (Scale bar: 2X = 1 mm, 20X = 100 μm). The above experiments were conducted twice and similar results were obtained. The representative data of one experiment are shown. Data represent the mean ± standard deviation of 4 hamsters at each time point in each group. The asterisks denote statistical differences between the indicated groups. n.s. no statistical difference, *P < 0.05; **P < 0.01; ***P < 0.001; ****P < 0.0001

To evaluate the immunogenicity of VRP-S-2P, different inoculation routes were performed in the mouse model which had been demonstrated to be sensitive to SARS-CoV-2 Omicron variant infection.^4^ Three doses of 1 × 10^6^ FFU VRPs were administered to cohorts of 6- to 8-week-old BALB/c mice with two weeks interval via intraperitoneal (IP), intramuscular (IM) and intranasal (IN) routes, respectively (Fig. 1d). VRP-eGFP encoding the eGFP gene and DMEM were used as negative controls. Specific anti-RBD IgG and neutralizing antibodies (NAbs) against the Omicron variant BA.1 were induced in all the immunized groups at 14 days after the last inoculation. Among them, the IP injection of the VRP-S-2P was most immunogenic that elicited the highest levels of IgG and NAbs, which was three/two times, and twenty-two/six times higher than those of IM and IN immunization, respectively (Fig. 1e, f). It has been reported that different administration routes are able to affect the balance of Th1 and Th2 immune responses.^5^ Vaccination is beneficial with a bias towards Th1-related response, since Th2-biased responses are associated with severe coronavirus diseases resulting from the vaccine-induced antibody-dependent enhancement.^6^ Here, we found that the VRP-S-2P IP-injected mice induced a Th1-biased antibody response with elevated ratio of IgG2a/IgG1, and differently, both IM and IN injection groups showed a slight Th2-biased response with low IgG2a/IgG1 ratios (Supplementary Fig. 1a–c). We think this phenomenon is related to the genetic background of BALB/c mice which are naturally preferred to Th2 response after immunization.

To evaluate the vaccine efficacy of VRP-S-2P, the immunized mice were intranasally challenged with 2 × 10^4^ PFU SARS-CoV-2 Omicron variant BA.1 at 49 days after the first immunization. At 3 dpi, the infected mice were sacrificed to measure the viral loads in lungs and nasal turbinates (Fig. 1g, h). Although there were similar low levels of virus loads in nasal turbinates of both vaccinated and DMEM-vaccinated groups, in contrast to high levels of viral loads detected in the lungs of VRP-eGFP and DMEM treated mice, no infectious viruses were detected in the VRP-S-2P IP and IM immunized mice, and 1Log_10_ decrease in viral titers was also observed in the VRP-S-2P IN immunized mice. These results demonstrated that VRP-S-2P immunization, especially via IP and IM routes, can effectively inhibit viral replication in lungs, conferring complete immunoprotective effect against Omicron variant in BALB/c mice.

Since the Omicron variant doesn’t cause lung damage in BALB/c mice, we extended the immunization experiments to Golden Syrian hamsters, which are more susceptible to the Omicron variant and exhibit obvious lung lesions after infection,^4^ to further assess the vaccine potential of VRP-S-2P. The same vaccination-challenge experiments were conducted in 4–6 weeks old female hamsters (Fig. 1i). As expected, all immunized hamsters, especially IP and IM immunized ones developed considerably high levels of RBD-specific IgG and NAbs against the Omicron variant BA.1 at 14 days after the last inoculation (Fig. 1j, k). Notably, VRP-S-2P, administrated through either route, elicited Th1 polarized antibody response in hamsters, as exhibited by elevated RBD-specific IgG2/3 titers (with the order of IP > IM > IN) and IgG1 levels as low as below the detection limit (Supplementary Fig. 2a, b). Furthermore, we found that VRP-S-2P-induced antibodies also exhibited sufficient neutralizing activities against SARS-CoV-2 prototype strain and other Omicron variants BA.2, BA.4 and BA.5, although the NAbs titers were 17.8-, 2.6-, 15.9- and 17.0-fold lower respectively than that against the Omicron BA.1 virus (Fig. 1k, supplementary Fig. 3).

At 3 days post-challenge, the lungs and nasal turbinates were collected for viral load quantification by plaque assay (Fig. 1l, m). In IP vaccinated group, no infectious virus was detected in either lungs or nasal turbinates. For IM group, there was a significant decrease of infectious virus production with average titers of 675 and 163 PFU/g in lung and nasal turbinate, respectively, which were 30,575 and 1387 PFU/g lower than those of the hamsters from DMEM treated group (31,250 PFU/g and 1550 PFU/g). The IN administration showed partial protection similar to that of IM route.

Pulmonary lesions caused by virus infection were observed on day 5 post-challenge. In spite of no significant weight changes during the whole observation period in both DMEM- and VRP-S-2P-vaccinated hamsters upon virus challenge (supplementary Fig. 4), visual observation results showed that apparent focal or multifocal dark red discoloration in the lung lobes occurred in the VRP-eGFP and DMEM treated groups, whereas no lung lesions were observed in any VRP-S-2P-immunized hamster (Fig. 1n). Moreover, histopathological analysis showed the hamsters from VRP-eGFP and DMEM groups developed severe interstitial pneumonia (Fig. 1o), with manifestations in inflammatory cell infiltration, eosinophilic secretions, perivascular edema with lymphocyte infiltration or tissue exudate, focal lymphoid infiltration, alveolar hemorrhage, lymphocytes infiltration in a sleeve shape and necrotic exfoliated epithelial cells. In contrast, only mild lymph focal infiltration and pulmonary edema were observed in the lungs of VRP-S-2P-immunized hamsters. Collectively, these results demonstrated that VRP-S-2P can robustly inhibit viral replication and completely protect hamsters against pulmonary lesions induced by Omicron variant.

In summary, our studies in mice and hamsters demonstrated that IP, IM, and IN delivery of VRP-S-2P vaccine confers protection against the SARS-CoV-2 Omicron variant BA.1 challenge. Protection is mainly associated with lower levels of viral loads and lesions remission in lungs. These findings support the application of alphavirus replicon particles delivering self-replicating RNA in the prophylactic vaccine development against the SARS-CoV-2 Omicron variant.

## Supplementary information

Supplementary materials

## Data Availability

All data supporting the findings of this study are available within the article or from the corresponding author upon reasonable request.
