# Supplementary material for: An alphavirus replicon particle delivering prefusion-stabilized spike protein provides potent immunoprotection against SARS-CoV-2 Omicron variant
Source: Signal Transduct Target Ther. 2022 Dec 14;7:390. doi: 10.1038/s41392-022-01246-x (PMC9747704; doi:10.1038/s41392-022-01246-x)
Supplement: Supplementary file 1 — Supplementary materials [file 41392_2022_1246_MOESM1_ESM.docx]

**Supplementary Materials for**

**An alphavirus replicon particle delivering prefusion-stabilized spike protein provides potent immunoprotection against SARS-CoV-2 Omicron variant.**

Hong-Qing Zhang^1,2^†, Ya-Nan Zhang^1^†, Zhe-Rui Zhang^1^†, Xiao-Ling Chen^1,2^, Yan-Yan Hu^1,2^, Yu-Jia Shi^1,3^, Jing Wang^1,2^,Cheng-lin Deng^1^, Bo Zhang^1,2*^, Xiao-Dan Li^1,3*^, Han-Qing Ye^1,2*^

^1^ Key Laboratory of Special Pathogens and Biosafety, Wuhan Institute of Virology, Center for Biosafety Mega-Science, Chinese Academy of Sciences, Wuhan, China;

^2^ University of Chinese Academy of Sciences, Beijing 100049, China;

^3^ Hunan Normal University, School of Medicine, Changsha, 410081, China

†These authors have equal contribution to this study

*Corresponding author: Han-Qing Ye, *yehq@wh.iov.cn*; Xiao-Dan Li: [*lxd@live.cn*](mailto:lxd@live.cn); Bo Zhang*:* [*zhangbo@wh.iov.cn*](mailto:zhangbo@wh.iov.cn)

**This PDF file includes:**

Materials and Methods

Supplementary Figures. 1 to 4

**Materials and Methods**

Cell lines, viruses and antibodies.

Vero-E6 cells (ATCC CRL-1686) and Baby hamster kidney (BHK-21) cells were maintained in Dulbecco’s modified Eagle’s medium (DMEM, Gibco) supplied with 10% (v/v) fetal bovine serum (FBS, Gibco), 100 U/mL penicillin and 100 μg/mL streptomycin (Beyotime). The Omicron variant BA.1 (CCPM-B-V-049-2112-18) was isolated from pharyngeal swab of infected patients in Hong Kong by the Institute of Experimental Animals, Chinese Academy of Medical Sciences. The Omicron variant BA.2 (CSTR: 16533.06.IVCAS 6.7617), BA.4 (CSTR: 16533.06.IVCAS 6.8980) and BA.5 (CSTR: 16533.06.IVCAS 6.8981) were provided by Microorganisms & Viruses Culture Collection Center, Wuhan Institute of Virology, Chinese Academy of Sciences. SARS-CoV-2 prototype strain (WIV04) was originally isolated from a COVID-19 patient in Wuhan. The SARS-CoV-2 prototype strain, Omicron variants BA.1, BA.2, BA.4 and BA.5 were propagated and titrated by plaque assays using Vero-E6 cells. All obtained viruses were stored aliquots at −80℃ and sequenced for experiments. Anti-SARS-CoV-2 RBD monoclonal antibody and Anti-SARS-CoV-2 S antibody were kindly provided by Prof. Bing Yan (Wuhan Institute of Virology, Chinese Academy of Sciences, Wuhan, China) and Prof. Shuo Shen (Wuhan Institute of Biological Products Co. Ltd, Wuhan, China), respectively.

Construction of a VEEV replicon expressing prefusion-stabilized spike protein of SARS-CoV-2 Omicron variant.

The cDNA clone of VEEV replicon with the entire deletion of the structural protein genes based on the backbone of the TC-83 strain was used for expressing the spike protein of SARS-CoV-2 Omicron variant (GenBank: UTN54706.1). Two proline substitutions were introduced into Omicron spike (S-2P) to stabilize its prefusion state. The S-2P full-length sequence was amplified and cloned into pACYC-VEEV replicon by unique restriction enzyme *Asc* I and *Pac* I. Primers introducing the mutations were designed with mutated overlapping nucleotides. In parallel, a VEEV replicon-control carrying an eGFP cassette was also generated.

The cDNA clones of two helper RNAs were constructed as our previous study described, encoding the capsid and envelop proteins of TC-83 strain, respectively [44]. All the plasmids used for virus replicon particles (VRP) production in the subsequent experiments were sequenced and verified.

Production and titration of VRP expressing Omicron S-2P (VRP-S-2P).

The cDNA clones of above replicon were linearized with *Not* I restriction enzyme and purified by phenol/chloroform extraction. The corresponding RNAs were transcribed and capped *in vitro* by using the mMESSAGE mMACHINE^TM^ T7 Transcription Kit (Invitrogen) according to the manufacturer's protocols. To generate the VRP-S-2P and VRP-eGFP, 10 μg of each replicon RNA and two helper RNA were co-electroporated into 1.2×10^7^ BHK cells in a 0.4 cm cuvette with a GenePulser apparatus (Bio-Rad) at 850 V and 25 μF. Three pulses were executed with 3 second intervals. The transfected-cells were placed at room temperature for 10 min, resuscitated with pre-heated medium in a T75 flask, and cultured at 37℃. After incubation for 24 h, cell supernatant was harvested, centrifuged, and filtered by 0.2 μm membrane. The resulting VRPs were stored aliquots at -80℃ for subsequent experiments.

The viral titration was determined by focus forming assay, which expressed as focus forming unit (FFU) per mL. Briefly, VRPs were 10-fold diluted and incubated with BHK cell slides (1×10^5^ per well) in 24-well plates at 37℃. After adsorption for 1 h, VRP dilutions were replaced with fresh medium and cultured for another 24 h. The slides were collected and analyzed for VRPs titration by indirect immunofluorescence assay.

Indirect immunofluorescence assay (IFA).

The BHK-21 cells were seeded on a Chamber Slide (Nalge Nunc) in 6-well plate. After adherence for 1 days, the cells were transfected or infected. At time points of sample collection, the cells were fixed with cold (−20 °C) 5% acetone in methanol at room temperature for 10 min and washed three times with PBS. For the detection of replicon replication, the cells were incubated with the primary antibody against RBD of SARS-CoV-2 for 1 h, followed by fluorescence labeling with the secondary antibodies FITC-conjugated goat anti-mouse IgG at room temperature for 1 h. The nuclei were stained with DAPI for 10 min. Following by PBS washing, the slides were mounted with 95% glycerol and analyzed under a Zeiss fluorescence microscope.

Western blotting assay (WB).

BHK cells were transfected with the transcribed RNAs, or infected with VRPs at a multiplicity of infection (MOI) of 1. At 24 hpi/t, the cells were lysed in RIPA buffer (Beyotime) on ice for 10 min, followed by denaturation at 95℃ for another 15 min. The samples were analyzed by SDS-PAGE electrophoresis and transferred onto 0.2 μm PVDF membrane (Bio-Rad). PVDF membrane was blocked by 5% skimmed milk for 2 h, and treated with primary antibody against SARS-CoV-2 S for 1 h. Then, PVDF membrane was incubated with secondary horseradish peroxidase (HRP)-conjugated anti-rabbit IgG (H+L) antibody. The signals were detected with a chemiluminescence system (Chemi-Doc, Bio-Rad) using Immobilon western chemiluminescent HRP substrate (Millipore).

Plaque assay.

Virus titration of SARS-CoV-2 in the supernatants of infected-cells and homogenized tissue was performed by monolayer plaque assay as described previously. Briefly, 1×10^5^ Vero-E6 cells per well were seeded into 24-well plate and then incubated with serially 10-fold diluted SARS-CoV-2 supernatant for 1 h at 37℃. After inoculation, virus supernatant was removed and the monolayer were overlaid with a DMEM mixture consisting of 0.8% methylcellulose, 2% FBS, 100 U/mL penicillin and 100 μg/mL streptomycin. The monolayer was cultured for 5 days to promote clear plaque formation, fixed with 3.7% formaldehyde for 24 h and stained with 1% crystal violet for 10 min. Plaques were counted after flushing with water.

Mice immunization and challenge.

Female BALB/c mice aged 6-8 weeks were divided into five groups (n=4). Cohorts of mice received 1×10^6^ FFU VRP-S-2P via intraperitoneal, intramuscular and intranasal routes, respectively. Meanwhile, the negative control of VRP-eGFP and the DMEM were also set as negative controls. Three immunizations were conducted with two-week intervals. At day 42 after the first inoculation, blood samples were collected from the orbit of mice, and heat-inactivated at 56℃ for 30 min. The total RBD-specific IgG and neutralizing antibodies against Omicron variant were measured by enzyme-linked immunosorbent assay (ELISA) and plaque reduction neutralization test (PRNT).

At day 49 after the first inoculation, all mice were intranasally challenged with 2×10^4^ PFU SARS-CoV-2 Omicron variant BA.1. Mice were monitored daily and sacrificed at 3 days post challenge, while the nasal turbinates and left lung were ground in DMEM. Infectious viral titer were quantified by plaque assay.

Golden Syrian hamsters immunization and challenge.

Hamsters, as a more susceptible model of SARS-CoV-2 infection, were used to further evaluate the immunogenicity and immunoprotection of VRP-S-2P. Female Golden Syrian hamsters aged 4-6 weeks were divided into five groups (n=8). Groups of hamsters received 1×10^6^ FFU VRP-S-2P via intraperitoneal, intramuscular and intranasal routes, respectively. Meanwhile, the negative control of VRP-eGFP and the DMEM were also set. The strategies for immunization and serological analysis were similar to those of mice.

At day 49 after the first inoculation, all hamsters were intranasally challenged with 2×10^4^ PFU SARS-CoV-2 Omicron variant BA.1. Clinical symptom and weight changes were observed daily. At 3 days after challenge, hamsters were sacrificed, along with the anatomy of the nasal turbinates and left lung, which was further homogenized for infectious virus quantification. The appearance of lung lesions were recorded, and the right lungs were fixed by 4% paraformaldehyde for 24 h, followed by pathologic analysis by H&E staining.

Enzyme-linked immunosorbent assay (ELISA)

The recombinant RBD protein (0.2 μg/mL) of Omicron BA.1 in 0.5M Na_2_CO_3_-NaHCO_3_ buffer was coated on 96-well plates overnight at 4℃. The plates were then blocked by 5% skimmed milk at 37℃ for 2 h. Sera from immunized mice and hamsters were 4-fold diluted with 2% skimmed milk and added to plates for 2 h incubation. After washing steps, HRP-conjugated goat anti-mouse/hamster IgG (H+L) antibody (1:5000 dilution) was added for 1 h at 37℃. The plates were incubated with a mixed substrate (two-component 3,3’,5,5’-tetramethylbenzidine (TMB) color development kit, Beyotime Biotechnology), and the optical density at 450 nm is detected and analyzed by a multimode microplate reader (Varioskan Flash; Thermo Fisher) after the addition of 1 M H_2_SO_4_ to terminate coloration reaction. The IgG antibody titers were defined as the highest dilution of sera giving an optical density twice than that of the naïve serum. Isotype determination of the antibody responses was performed using HRP-conjugated isotype-specific (IgG1 or IgG2a) goat anti-mouse antibodies (1070-05 and 1080-05, SouthernBiotech, 1:8000) and HRP-conjugated isotype-specific (IgG1 or IgG2/3) mouse anti-hamster antibodies (1940-05 and 1935-05, SouthernBiotech, 1:8000).

Plaque reduction neutralization test (PRNT).

Sera from immunized mice and hamsters were 10-fold diluted with DMEM medium and incubated with 50 PFU SARS-CoV-2 prototype strain or Omicron variants BA.1/BA.2/BA.4/BA.5. The serum-virus mixture was pre-incubated at 37℃ for 1 h, and added to the Vero-E6 monolayer in 24-well plates. After 1 h adsorption, the monolayer was overlaid with a DMEM mixture consisting of 0.8% methylcellulose, 2% FBS, 100 U/mL penicillin and 100 μg/mL streptomycin. The monolayer was cultured for 5 days to promote clear plaque formation, fixed with 3.7% formaldehyde for 24 h and stained with 1% crystal violet for 10 min. Serum neutralization titers were defined as the highest dilutions given a 50% plaque reduction (PRNT_50_) than that of the naïve serum.

Statistical Analysis

All data were analyzed using GraphPadPrism 8.0.2 software and expressed as mean ± standard deviation (SD). The statistical significance was assigned when P values were < 0.05. Student’s T-test was used to analyze the differences between two groups, and significant differences between groups were determined using a two-way analysis of variance (ANOVA).

**Supplementary Figures.**


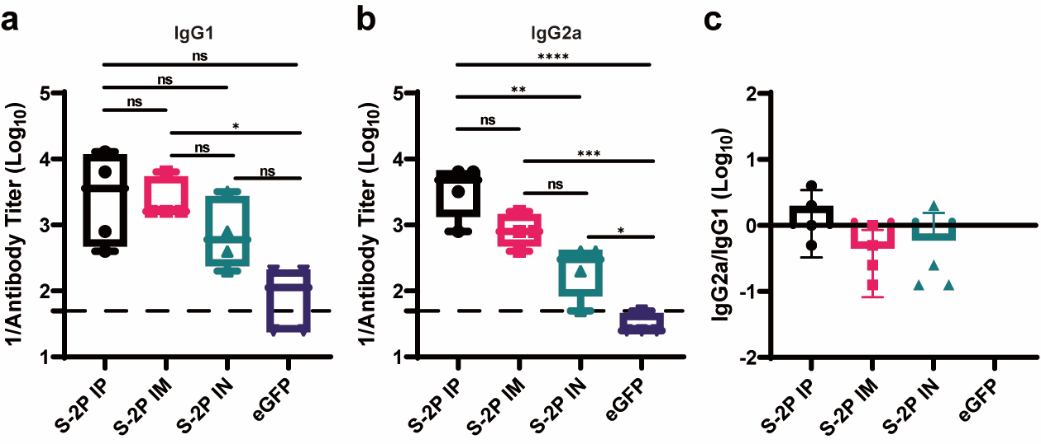


**Supplementary Figure. 1 Isotype antibodies determination in the sera of mice immunized with VRP-S-2P.** Female BALB/c mice aged six- to eight- weeks were immunized with 1×10^6^ FFU VRP-S-2P via intraperitoneal, intramuscular and intranasal routes, respectively. Three immunizations were conducted with two-week intervals. On day 42 post immunization, serum samples were collected. Titers of subtype IgG1 (**a**) and IgG2a (**b**) were measured by enzyme-linked immunosorbent assay (ELISA). (**c**) Ratios of IgG2a and IgG1 were analyzed to identify the preference of Th responses. Dashed lines represent the limit of detection. n.s., no statistical difference, *, P<0.05; **, P<0.01; ***, P<0.001; ****, P<0.0001.


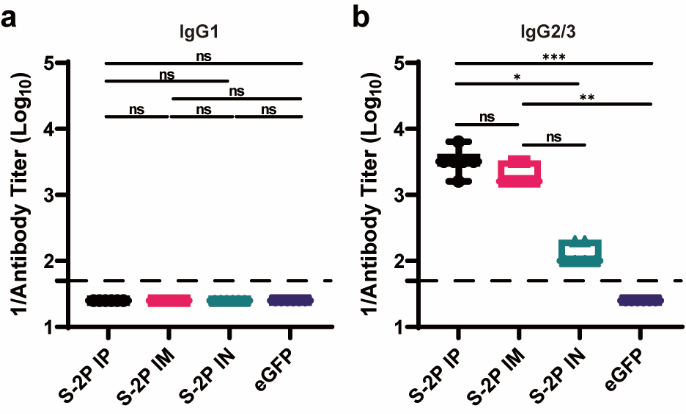


**Supplementary Figure. 2 Isotype antibodies determination in the sera of hamsters immunized with VRP-S-2P.** Female Golden Syrian hamsters aged four- to six- weeks were immunized with 1×10^6^ FFU VRP-S-2P via intraperitoneal, intramuscular and intranasal routes, respectively. Three immunizations were conducted with two-week intervals. On day 42 post immunization, serum samples were collected. Titers of subtype IgG1 (**a**) and IgG2/3 (**b**) were measured by enzyme-linked immunosorbent assay (ELISA). n.s., no statistical difference, *, P<0.05; **, P<0.01; ***, P<0.001; ****, P<0.0001.


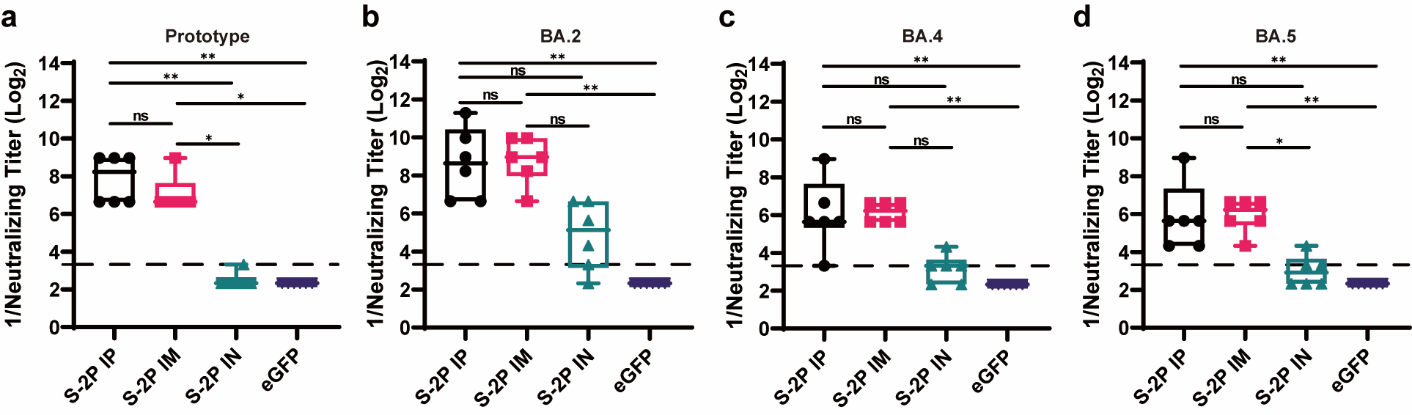


**Supplementary Figure. 3 Neutralizing antibodies detection against SARS-CoV-2 prototype and Omicron sublineage BA.2, BA.4 and BA.5 in the sera of hamsters immunized with VRP-S-2P.** Female Golden Syrian hamsters aged four- to six- weeks were immunized with 1×10^6^ FFU VRP-S-2P via intraperitoneal, intramuscular and intranasal routes, respectively. Three immunizations were conducted with two-week intervals. On day 42 post immunization, serum samples were collected. Neutralizing antibodies against SARS-CoV-2 prototype (**a**) and Omicron sublineage BA.2 (**b**), BA.4 (**c**) and BA.5 (**d**) were measured by PRNT. n.s., no statistical difference, *, P<0.05; **, P<0.01; ***, P<0.001; ****, P<0.0001.

**
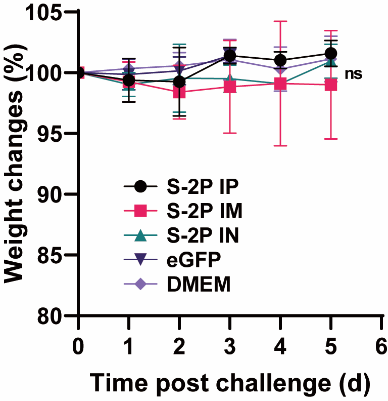
**

**Supplementary Figure. 4 Weight changes of the immunized hamsters after challenge with Omicron variant BA.1.** At 49 days after first inoculation, the immunized hamsters were challenged with 2×10^4^ PFU Omicron variant BA.1. The weight changes of the immunized hamsters were recorded daily during 5 days post challenge. n.s., not significant.
